# Plant Interactions Alter the Predictions of Metabolic Scaling Theory

**DOI:** 10.1371/journal.pone.0057612

**Published:** 2013-02-27

**Authors:** Yue Lin, Uta Berger, Volker Grimm, Franka Huth, Jacob Weiner

**Affiliations:** 1 Institute of Forest Growth and Computer Science, Dresden University of Technology, Tharandt, Germany; 2 Helmholtz Centre for Environmental Research – UFZ, Department of Ecological Modelling, Leipzig, Germany; 3 Institute for Biochemistry and Biology, University of Potsdam, Potsdam, Germany; 4 Institute of Silviculture and Forest Protection, Dresden University of Technology, Tharandt, Germany; 5 Department of Plant and Environmental Sciences, University of Copenhagen, Frederiksberg, Denmark; University of Zurich, Switzerland

## Abstract

Metabolic scaling theory (MST) is an attempt to link physiological processes of individual organisms with macroecology. It predicts a power law relationship with an exponent of −4/3 between mean individual biomass and density during density-dependent mortality (self-thinning). Empirical tests have produced variable results, and the validity of MST is intensely debated. MST focuses on organisms’ internal physiological mechanisms but we hypothesize that ecological interactions can be more important in determining plant mass-density relationships induced by density. We employ an individual-based model of plant stand development that includes three elements: a model of individual plant growth based on MST, different modes of local competition (size-symmetric vs. -asymmetric), and different resource levels. Our model is consistent with the observed variation in the slopes of self-thinning trajectories. Slopes were significantly shallower than −4/3 if competition was size-symmetric. We conclude that when the size of survivors is influenced by strong ecological interactions, these can override predictions of MST, whereas when surviving plants are less affected by interactions, individual-level metabolic processes can scale up to the population level. MST, like thermodynamics or biomechanics, sets limits within which organisms can live and function, but there may be stronger limits determined by ecological interactions. In such cases MST will not be predictive.

## Introduction

Metabolic Scaling Theory (MST) offers a quantitative framework for linking physiological processes of individual organisms with higher-level dynamics of populations and communities. It predicts that an individual’s metabolic rate, *B*, scales with body mass, *m*, as *m* ^3/4^ [1]. For plants, it is assumed that *B* is proportional to their rate of resource use, *Q*, and increases with body mass, *m*, as *B*∝*Q*∝*m* ^3/4^ [2]. When the rate of resource supply, *R*, per unit area is held constant, the relationship between maximum population density, *N*, and mean body mass is predicted to be *m*∝*N* ^−4/3^. Thus, if mass-density relationships during self-thinning reflect MST, the relation between *m* and *N* is predicted to be a power law with a mass–density scaling exponent of –4/3.

While broad interspecific patterns are sometimes consistent with this prediction [2], [3], the empirical observations from self-thinning populations are more variable [4]–[7]. Data from arid regions or areas with low resource levels often deviate from the predictions of MST and show significantly shallower trajectories, i.e. less negative exponents [4], [7]. While some researchers assume that the mass–density scaling exponent is universal but disagree about the correct value, others argue that there is real biological variation in the exponent, thus questioning the generality of MST [4], [8]–[10] or any other single model which purports to explain many different types of biomass-density relationships.

A core assumption of MST is that processes internal to individuals determine mass-density relationships. An alternative view is that internal mechanism may play an important role and set limits on mass-density relationships, but that ecological interactions can be more important in determining the relationships in the field. Thus, variation in the ecological conditions can explain the observed variation in scaling exponents. Specifically, it has been argued that competition among plants will change mass-density relationships from those predicted by MST [7], [9]. The well-documented plasticity of plant form in response to competition [5], [11] suggests that competitive interactions could affect mass-density relationships.

Many empirical studies on plant mass-density relationships have based on data where competition for light dominates [2], [4], [12], but in areas where below-ground resources such as nutrients and water are more limiting canopies can remain unclosed. In such areas below-ground competition may affect growth and mortality much more than above-ground competition [4], [7], [13].

Below- and above-ground competition are qualitatively different. Above-ground, the limiting resource, light, is directional and therefore “pre-emptable”, i.e. taller plants will have a disproportionate advantage over smaller individuals when competing for light, which has also been referred to as “size-asymmetric competition”, “dominance and suppression” or “one-sided competition” [14]–[16]. In contrast, below-ground resources such as water and nutrients are not generally pre-emptable so that competing plants tend to share below-ground resources in proportion to their sizes. There is much evidence that above-ground competition tends to be size-asymmetric, while below-ground competition is more size-symmetric [14]–[16]. This could influence mass-density relationships.

There is evidence to support this claim. For example, for the desert shrub *Larrea tridentata*, the individuals’ allometric growth and root-shoot biomass allocation patterns are consistent with MST, but the log mass - log density relationship is shallower than predicted by MST with a substantial variation [13]. This suggests that below-ground competition, which is more size-symmetric, may leads to shallower self-thinning trajectories. Results from an individual-based zone-of-influence plant population model indicate that the size-symmetry or asymmetry of competition will affect self-thinning trajectories [15], [17]. These studies used a phenomenological model for individual plant growth [18] that does not accommodate the physical and biological principles of MST. And indeed, the range of slopes produced by Chu *et al*.’s model [17], from −0.820 to 1.609, is larger than the range observed in the field. For example, in 1266 plots within six biomes and 17 forest types across China, the estimated log mass - log density slopes ranged from −1.103 to −1.441 [19].

We hypothesize that MST may be compatible with the observed variation in self-thinning trajectories if different modes of competition and different resource availabilities are considered. We investigate two hypotheses:

1. Size-symmetric competition (e.g. below-ground competition) will lead to shallower self-thinning trajectories.
2. Individual-level metabolic processes can predict population-level mass-density relationships if surviving plants are not highly affected by local interactions.

To investigate our hypothesis, we modify a widely used individual-based zone-of-influence model of individual growth and competition, in which competition can be size-symmetric or -asymmetric [18]. To make our model compatible with the assumptions of MST, we use an individual growth model and allometric relationships derived from MST [20].

## Methods

### The Model

The individual plant growth model used here is similar to that described previously [20], which was based on an energy conservation equation [12], [21], [22]. It takes into consideration three basic processes that require energy: maintenance of biomass, ion transport and biosynthesis [23]. Using empirical measurements and theoretical assumptions, MST predicts quantitative relationships among these processes [3], [24], and we use these as the basis of our individual growth model for plants:
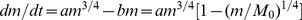
(1)where *m* is the plant’s total biomass and *a* and *b* species-specific constants (File S1). Our derivation of this model is similar to the derivation of growth models for animals [21], [22]. The value of *M*_0_ = (*a*/*b*)^4^ is the asymptotic maximum body mass of plant (calculated for *dm*/*dt* = 0), which depends on species-specific traits and is determined by the systematic variation of the in *vivo* metabolic rate within different taxa [21]. The gain term (*am* ^3/4^) in equation (1) dominates early in plant growth, and has some empirical support [24], [25]. Equation (1) is similar to the “von Bertalanffy growth model”, but its derivation here is based on physical and biological principles of MST [21], [22].

In our spatially explicit, individual-based model [17], plants are modelled as circles growing in 2-dimensional space [18]. The area of the circle, *A*, represents the resources available to the plant, and this area is allometrically related to the plant’s body mass, *m*, as *m* ^3/4^ = *c*_0_ *A* [12], where *c*_0_ is a normalization constant. Plants compete for resources in areas in which they overlap, and the mode of competition is reflected in the rules for dividing up the overlapping areas. Resource competition is incorporated by using a dimensionless competition index, *f_p_*, whose value is determined by the overlap with neighbors, by reducing the resources available in area *A*. With these assumptions, equation (1) becomes:
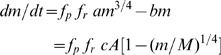
(2)where *M* = (*f_p_f_r_*)^4^ *M*_0_ represents maximum achievable biomass under resource limitation and competition, and where *c* = *ac*_0_ is the initial growth rates in units of mass per area and time interval. We represent resource limitation with a dimensionless efficiency factor, *f_r_*, as different levels of resource availability. For simplicity, we use a linear form here, i.e. *f_r = _*1*– RL*, where *RL* indicates the level of resource limitation, and ranges from 0 (no resource limitation) to 1 (maximum resource limitation; File S1).

The mode of resource-mediated competition among plants can be defined anywhere along a continuum from completely size-asymmetric competition (the largest plants obtain all the contested resources) to completely symmetric competition (resources in areas of overlap are divided equally among all overlapping individuals, independent of their relative sizes) [14]. To represent the different modes of competition explicitly, we define the effect of competition, *f_p_*, as
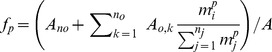
(3)


This index refers to the fraction of resources available in the ZOI which the plant *i* could obtain after a loss of potential resources due to areas overlapped by *n_j_* individuals of sizes *m_j_* [14]. *A_no_* is the area not overlapping with any neighbors, and *A_o,k_* indicates the area overlapped by neighbors. Parameter *p* determines the mode of competition, ranging from complete symmetry (*p* = 0) to complete asymmetry (*p* = ∞).

In MST, individuals’ mortality rate is assumed to be proportional to their mass-specific metabolism [25]. Based on this, we assume that individuals die if their actual growth rate (realistic metabolic rate) falls below a threshold fraction of their basal metabolic rate (scaled by current biomass, i.e. 2% of *m* ^3/4^). Therefore, individual plants may die due to metabolic inactivation driven by resource limitation, competition, senescence (when *m* approaches *M*) or combinations thereof. A detailed model description following the ODD protocol (Overview, Design concepts, Details) for describing individual- and agent-based models [26], [27] is provided in the Supplementary Material (File S1), as well as its implementation in NetLogo 3.1.4 [28] (File S2).

### Simulations and Analysis

In our simulations, we investigated 4 resource limitation levels (*RL* equal to 0, 0.1, 0.5 and 0.9), 4 modes of competition (*p* = ∞: completely asymmetric; *p = *10: highly size-asymmetric; *p = *1: perfectly size-symmetric; *p = *0: completely symmetric) and one initial density (8,100 individuals per total area). We also investigated other initial densities and the results were very similar to those presented below. Simulations for the resulting 16 scenarios were repeated five times using different random initializations.

We used the relative interaction index RII [29] to evaluate the effects of local competition on shaping plant mass-density relationship:
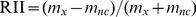
(4)where *m*_x_ and *m*_nc_ are the performance (mean biomass) of surviving plants at the same resource level with and without local competition (i.e., isolated plants), respectively. Values of RII from −1 to 1 indicate the intensity of interactions as competition (from −1 to 0), neutral interaction (equal to 0) and facilitation (from 0 to 1). To estimate *m*_nc_, we use the growth equation (2) without the competitive factor *f*_p_.

For linear fits of the self-thinning trajectories obtained with our model, we selected data points on the basis of mortality [30]: after density-dependent mortality starts, data points with surviving plants no less than 10% of the initial density (not less than 800 surviving plants here) and with the relative mortality larger than a threshold (the mean value of relative mortality at each time step through self-thinning process) were selected to fit the self-thinning trajectories. The thinning trajectories (log-log transformed data of mean biomass vs. density of survivors) were fitted by reduced major axis (type II model) regression, which assumes error in both variables and is widely used to investigate mass-density relationships. All statistical analyses were conducted using R 2.11.1.

## Results

**Figure 1:**
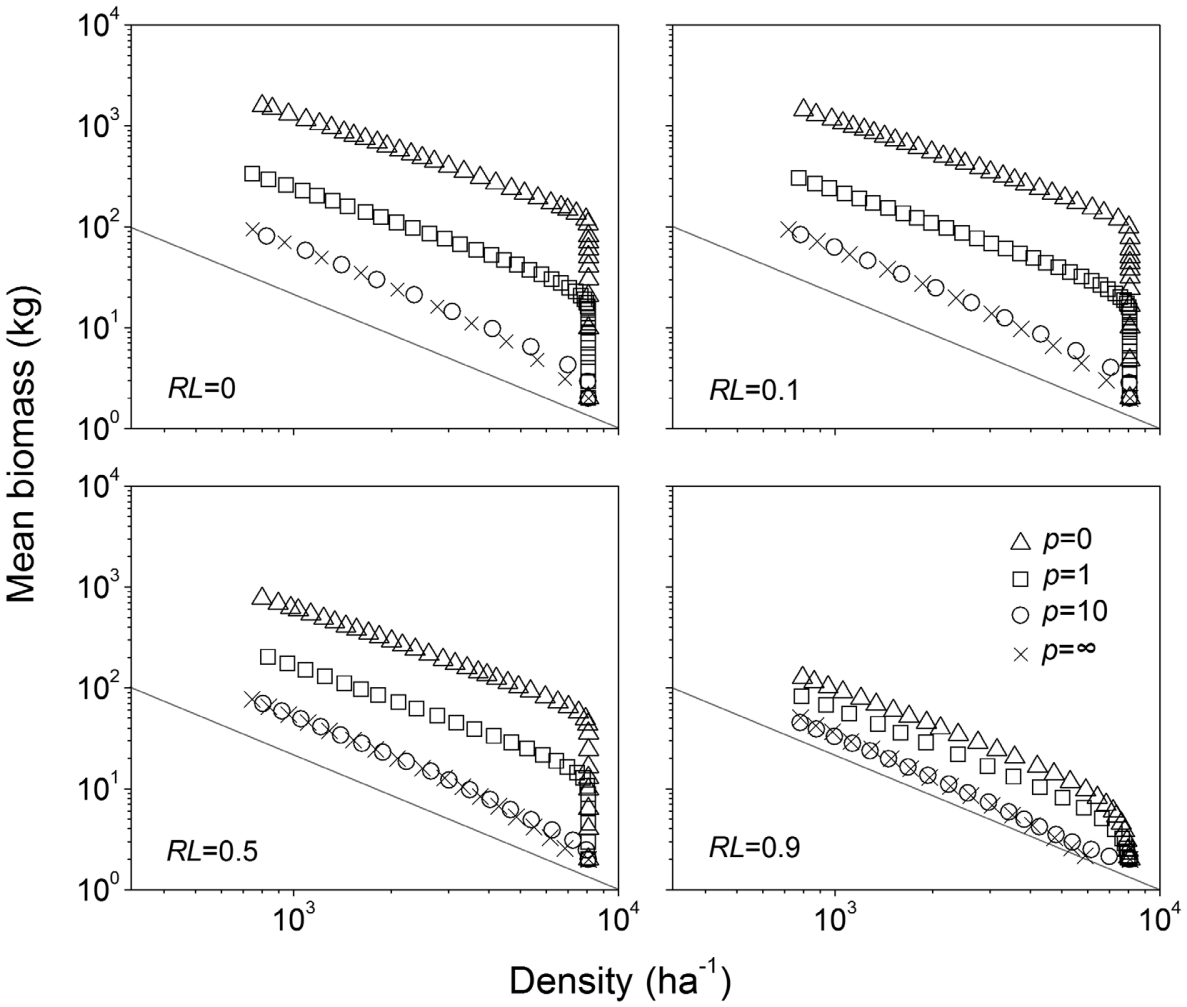
Self-thinning trajectories for different levels of resource limitation and modes of competition. *RL* indicates the level of resource limitation (from 0 to 1 indicating no limitation to extreme limitation), *p* indicates the modes of competition (∞: completely asymmetric; 10: highly size-asymmetric; 1: perfectly size-symmetric; 0: completely symmetric). For comparison, the solid lines have slopes equal to−4/3.

**Figure 2:**
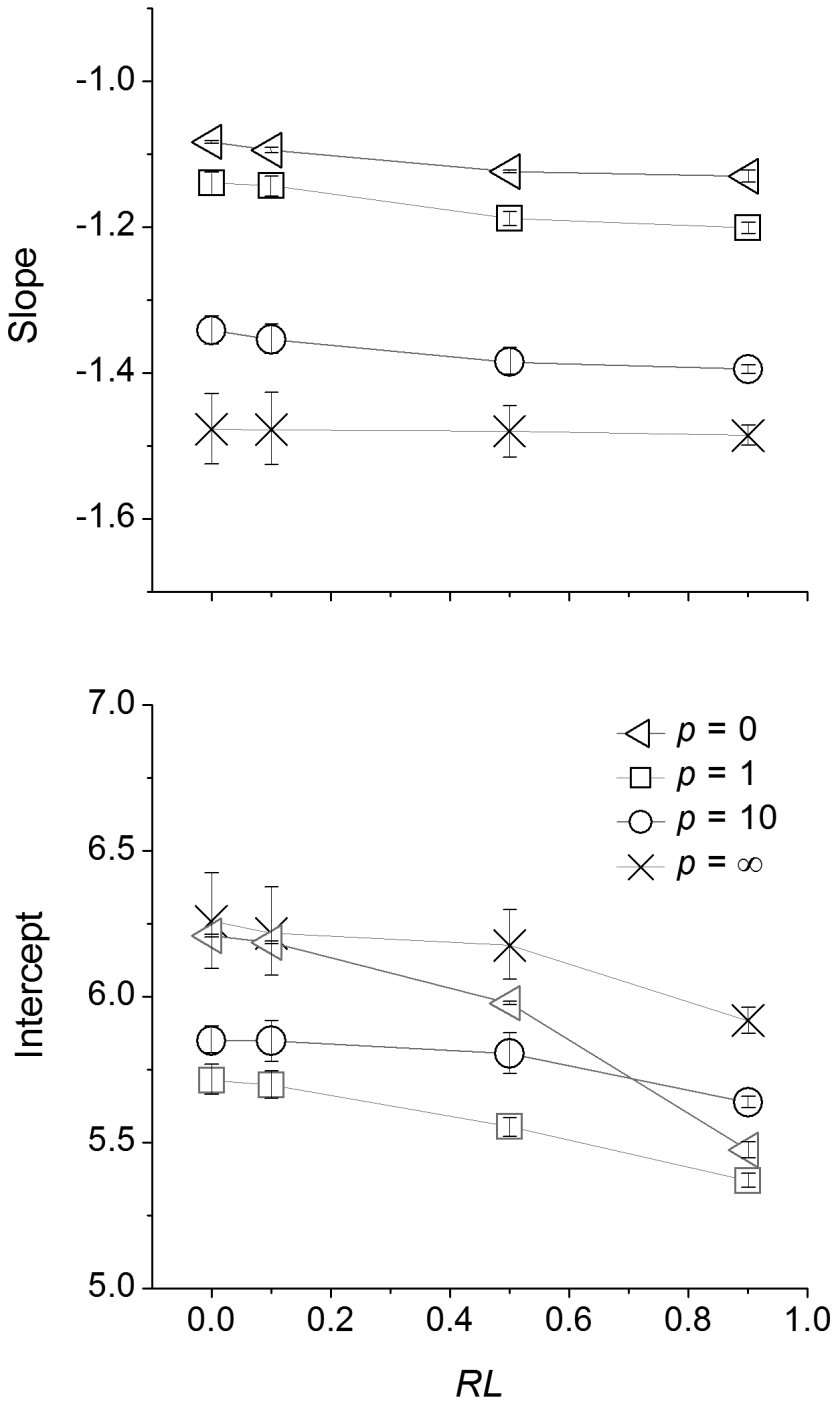
Slopes and intercepts of self-thinning trajectories of mean individual biomass versus survivor density under different levels of resource limitation and modes of competition. *RL* indicates the level of resource limitation (from 0 to 1 indicating no limitation to extreme limitation), *p* indicates the modes of competition (∞: completely asymmetric; 10: highly size-asymmetric; 1: perfectly size-symmetric; 0: completely symmetric). Bars indicate 95% confidence intervals.

Variation in the mode of competition, the level of resource limitation and their interaction produced significant variation in the self-thinning trajectory (Figure 1, Table S1). The mode of competition had a greater effect on the slope of self-thinning trajectories than did the level of resource limitation. For given resource limitation, *RL*, symmetric competition made self-thinning trajectories significantly shallower (ninety-five percent confidence intervals for the four modes of competition did not overlap), but within same mode of competition the level of resource limitation did not change slopes much (Figure 2).

**Figure 3:**
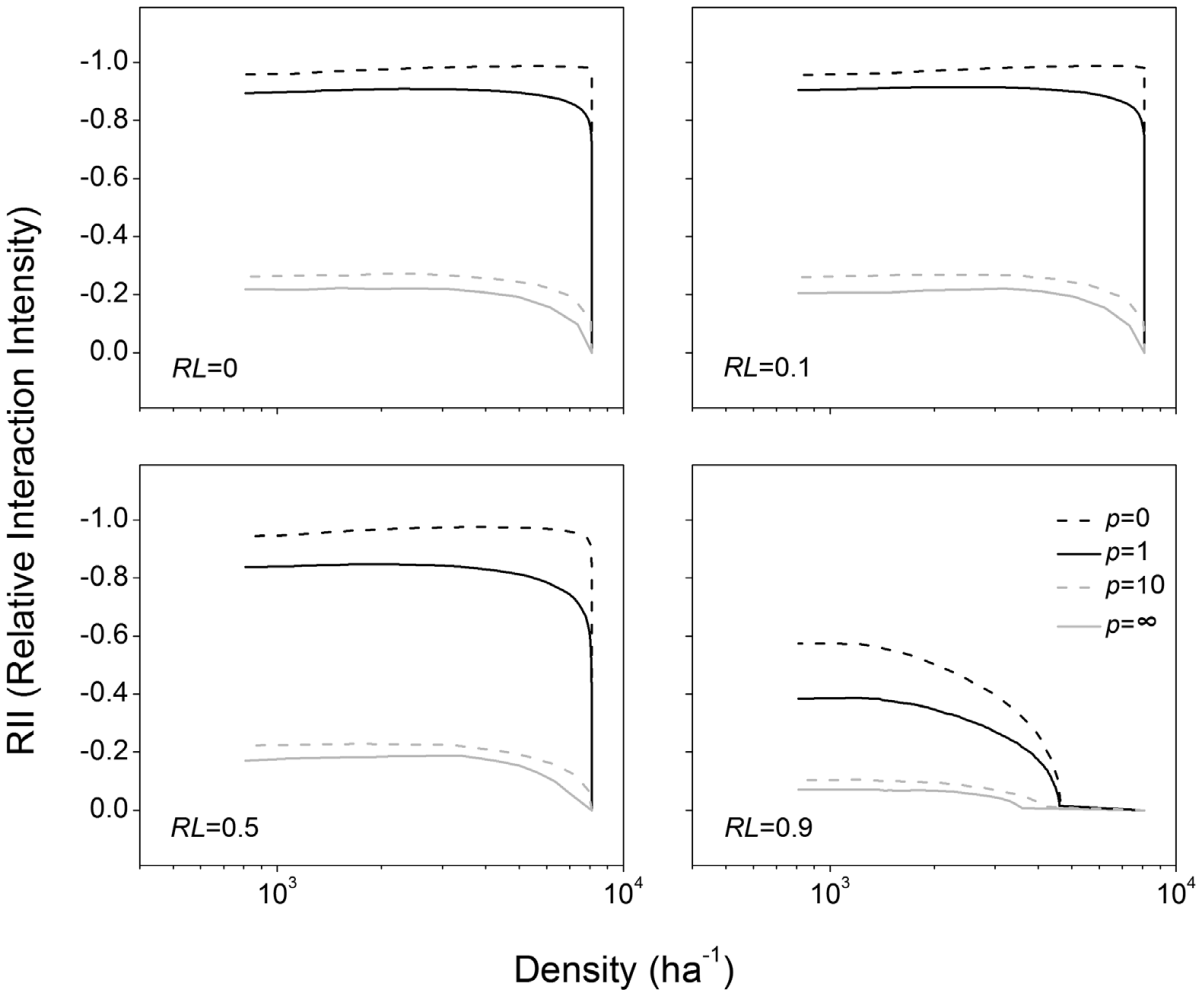
Relationship between relative interaction intensity (RII) and density of surviving plants at different levels of resource limitation and modes of competition. *RL* indicates the level of resource limitation (from 0 to 1 indicating no limitation to extreme limitation), *p* indicates the modes of competition (0: completely symmetric; 1: perfectly size-symmetric; 10: highly size-asymmetric; ∞: completely asymmetric). Values of RII indicate the intensity of interactions as competition (from −1 to 0), neutral interaction (equal to 0) and facilitation (from 0 to 1).

In scenarios with more symmetric competition, the relative interaction index RII is close to −1 and thus the effect of competition on surviving individuals is quite strong (Figure 3). In contrast, in scenarios with more asymmetric competition, surviving plants are less affected by interactions with other plants (RII close to 0). Both resource limitation and asymmetric competition lowered the position of self-thinning trajectories: less biomass can be accumulated at a given density under resource limitation or with more asymmetric competition (Figure 1). Resource limitation decreased intercepts within the same mode of competition (Figure 2), which means that the maximum biomass of plants is smaller in harsh conditions.

## Discussion

Our results and those of previous studies [15], [17] are consistent with our first hypothesis that symmetric competition can lead to shallower self-thinning trajectories than asymmetric competition. This suggests that deviations from the slope predicted by MST are likely to occur when competition below ground is stronger than above ground because the former is size-symmetric [14], [31]. Indeed, several empirical studies show that slopes of self-thinning trajectories are significantly flatter under severe water stress [4] and low nutrient levels [7], conditions in which competition below ground is thought to be more important than above-ground [4], [14], [16]. Furthermore, boreal coniferous forests tend to have steeper slopes than deciduous broadleaved forests because the canopy of conifers is denser (with lower light transmittance) suggesting that asymmetric competition for light is more intense [15], [19], [30].

Why does symmetric competition lead to flatter trajectories in our model even though it makes surviving plants larger on average at a given density (Figure 1)? With symmetric competition, the growth of all individuals is significantly reduced but the onset of mortality is delayed [18]. Plants can survive and grow even at relatively high densities, meaning that more biomass can be maintained at a given density when competition is size-symmetric [15].

When competition is highly size-asymmetric, surviving plants are less affected by their neighbors and thus individual-level metabolic processes of MST predict plant mass-density relationship at the population level: the slope of the self-thinning trajectories is close to −4/3. This is consistent with our second hypothesis that MST’s predictions of population-level mass-density relationships are successful when surviving plants are not strongly affected by local interactions. In stands of *Nothofagus solandri* (mountain beech), taller trees are relatively unhindered by competition for light and show the scaling of diameter growth which is consist with prediction of MST, whereas small trees affected by asymmetric competition do not follow the growth trajectory of prediction [8], [9]. In a tropical rainforest, trees under high light conditions are not highly affected by neighboring trees. Consistent with our second hypothesis, diameter growth of such individuals was consistent with the predictions of MST [10].

In contrast to our findings, Coomes and coworkers concluded that deviations from predictions of MST in forests are caused by size-asymmetric competition [8], [9]. On closer inspection, our results are not inconsistent with those of Coomes and coworkers. Coomes’s studies focused on diameter growth of individuals, including small suppressed individuals that are experiencing size-asymmetric competition for light, whereas we focus here on the mass-density relationship of populations during self-thinning, i.e. on plants surviving competition. When competition is highly size-asymmetric, total biomass is primarily due to the largest individuals, which are not highly affected by neighbors. If one looks at smaller individuals suffering from asymmetric competition before they die, however, they will be highly affected by their larger neighbors, and this will be more important for their growth and density than the internal relationships that form the basis for MST. It would be worthwhile to analyze individual-level diameter growth in our model to compare the patterns with those from empirical studies [8]–[10] to see if the conclusions are consistent. Nevertheless, we agree with Coomes and coworkers on the central point: interactions among individuals can overrule the predictions of MST.

Both size-asymmetric competition and resource limitation lowered self-thinning trajectories (Figure 2). Resource limitation reduces the growth of individual plants, leading to smaller individuals. Size-asymmetric competition results in faster mortality. The reduction of biomass due to mortality is not immediately compensated by the growth of survivors, so there is less total biomass at a given density.

Using a similar model, Chu *et al*. [17] found the same effect of the mode of competition (size symmetric vs. asymmetric) on the slope of self-thinning trajectories. The most important difference between Chu *et al*.’s model and ours is that we used an individual growth model that is derived from MST, whereas they used the phenomenological growth equation of Weiner *et al.* [18]. Chu *et al*. [17] focused on the effects of mode of competition, resource levels, and facilitation on self-thinning *per se*, so they do not refer to MST or focus on their choice of their individual growth model. This may be why the range of scaling exponent predicted with our model (−1.083 to −1.486) is closer to the observed range of exponents (−1.103 to −1.441 for 1266 plots of six biomes and 17 forest types across China) [19] than models using phenomenological growth functions (−0.8204 to −1.6095) [17]. This suggests that the consideration of both neighborhood interactions and constraints provided by MST are necessary to explain the biomass-density relationships observed in the field.

Our results point to the scope and limits of MST at the population and community level. MST applies to individual organisms, not always and necessarily to populations or ecosystems. In some cases, for example where resources are not limiting and competition is highly size-asymmetric, the mass-density scaling exponent predicted by MST matches observations quite well. This is because individual acquisition of resources and accumulation of biomass is driven primarily by what the individual itself does rather than by interactions with other individuals (Figure 3). On the other hand, when individual behavior is determined more by interactions with their neighbors rather than processes that are the bases of MST, the population-level behavior will deviate from the predictions of MST, as has been shown for Dynamic Energy Budget Theory [32]–[34].

### Conclusions

MST, like energetics and biomechanics, sets limits on the behavior of individuals and therefore of populations and communities. In some ecological situations these limits will dominate, and MST will predict higher-level behavior. In many cases however, other constraints are stricter, and these, rather than MST, will determine the patterns observed. Our most important conclusion is that the behavior of populations and communities may be dominated by internal physiological mechanisms addressed by MST or by ecological factors beyond the individual level, such as the type of resource limitation and the mechanisms of competition among individuals. In the latter cases MST will not be predictive, although nor will it be violated. The claim that MST provides a universal and mechanistic basis for quantitatively linking the energetic metabolism of individuals to ecological community dynamics is too strong. MST sets constraints within which ecology operates, but these are not always the dominant constraints. The observed variation in mass-density relationships represents variation in the most restrictive of the potential constraints in a given ecological context. Consideration of competition is critical for understanding variation in observed mass-density relationships.

## Supporting Information

Table S1 **Slope and intercept of self-thinning trajectories.**
(DOCX)

File S1 **ODD model description of pi model.**
(DOC)

File S2 **NetLogo file of pi model v1.3.**
(NLOGO)
