# Supplementary material for: Plant Interactions Alter the Predictions of Metabolic Scaling Theory
Source: PLoS One. 2013 Feb 27;8(2):e57612. doi: 10.1371/journal.pone.0057612 (PMC3584043; doi:10.1371/journal.pone.0057612)
Supplement: Table S1 — Slope and intercept of self-thinning trajectories. (DOCX) [file pone.0057612.s001.docx]

**Table S1.** Slope and intercept (log-log transformed) of self-thinning trajectories for simulated plants under different levels of resource limitation and modes of competition*.

| *RL* | *p* | Slope | | |  | Intercept | | | *R*^2^ |
| --- | --- | --- | --- | --- | --- | --- | --- | --- | --- |
|  |  | Mean | 95% C.I. | |  | Mean | 95% C.I. | |  |
| 0 | ∞ | -1.478 | -1.525 | -1.429 |  | 6.258 | 6.096 | 6.424 | 0.994 |
| 0 | 10 | -1.342 | -1.361 | -1.322 |  | 5.848 | 5.808 | 5.898 | 0.999 |
| 0 | 1 | -1.140 | -1.156 | -1.125 |  | 5.714 | 5.664 | 5.769 | 0.997 |
| 0 | 0 | -1.083 | -1.085 | -1.082 |  | 6.208 | 6.204 | 6.212 | 0.999 |
| 0.1 | ∞ | -1.478 | -1.526 | -1.427 |  | 6.217 | 6.074 | 6.377 | 0.994 |
| 0.1 | 10 | -1.354 | -1.374 | -1.333 |  | 5.848 | 5.778 | 5.918 | 0.998 |
| 0.1 | 1 | -1.144 | -1.158 | -1.130 |  | 5.697 | 5.650 | 5.744 | 0.997 |
| 0.1 | 0 | -1.094 | -1.098 | -1.091 |  | 6.185 | 6.180 | 6.190 | 0.999 |
| 0.5 | ∞ | -1.480 | -1.516 | -1.445 |  | 6.176 | 6.060 | 6.297 | 0.995 |
| 0.5 | 10 | -1.385 | -1.402 | -1.365 |  | 5.805 | 5.736 | 5.875 | 0.997 |
| 0.5 | 1 | -1.188 | -1.198 | -1.179 |  | 5.554 | 5.519 | 5.585 | 0.998 |
| 0.5 | 0 | -1.124 | -1.126 | -1.122 |  | 5.978 | 5.972 | 5.984 | 0.999 |
| 0.9 | ∞ | -1.486 | -1.499 | -1.472 |  | 5.918 | 5.873 | 5.963 | 0.996 |
| 0.9 | 10 | -1.395 | -1.401 | -1.389 |  | 5.637 | 5.619 | 5.657 | 0.999 |
| 0.9 | 1 | -1.201 | -1.209 | -1.194 |  | 5.370 | 5.345 | 5.395 | 0.998 |
| 0.9 | 0 | -1.130 | -1.139 | -1.122 |  | 5.474 | 5.446 | 5.502 | 0.997 |

**RL* indicates the level of resource limitation (0–1), *p* indicates the modes of competition (with 0: completely symmetric; 1: perfectly size-symmetric; 10: highly size-asymmetric; ∞: completely asymmetric). C.I. is confidence interval.
