# Supplementary material for: Plant Interactions Alter the Predictions of Metabolic Scaling Theory
Source: PLoS One. 2013 Feb 27;8(2):e57612. doi: 10.1371/journal.pone.0057612 (PMC3584043; doi:10.1371/journal.pone.0057612)
Supplement: File S1 — ODD model description of pi model. (DOC) [file pone.0057612.s002.doc]

**File S1**

**Plant interactions alter the predictions of metabolic scaling theory**

Yue Lin*, Uta Berger, Volker Grimm, Franka Huth and Jacob Weiner

* Institute of Forest Growth and Computer Science, Dresden University of Technology, Tharandt, Germany

yue.lin.tud@gmail.com

**ODD protocol - Model description**

**INDIVIDUAL-BASED MODEL (IBM) CONSIDERING MODE OF COMPETITION, RESOURCE LIMITATION AND ONTOGENETIC GROWTH**

The following model description follows the ODD protocol (Overview, Design concepts, Details) for describing individual- and agent-based models (Grim*m et* al. 2006; Grimm *et al*. 2010),

*Purpose*

The aim of this model is to evaluate the multiple effects of the mode of competition and resource limitation on regulating plant population dynamics, specifically on self-thinning trajectories and density-dependent mortality. In particular, we investigate whether interactions on individual plant level can alter the slope and intercept of the self-thinning line. The model does not represent specific species, but generic ones.

*Entities, state variables, and scales*

The entities in the model are plants and square habitat units, or patches (Table A1). Plants are characterized by the following state variables: initial growth rate, initial biomass, maximum biomass (asymptotic biomass), current biomass and their position, i.e. coordinates of the stem. Each individual plant has its own circular zone-of-influence (ZOI). The ZOI stands for the physical space occupied by a plant, and represents the energy and resources potentially available to this plant, which is allometrically related to its body mass. Neighboring plants only compete for the resources when their ZOIs are overlapping.

In order to make the spatial calculations of resource competition easier, ZOIs are projected onto a grid of patches. To avoid edge effects, we use a torus world with a size of 200 × 200 patches (Grimm & Railsback 2005). Each patch represents 0.25 m^2^ or 0.25 cm^2^ for woody- and non-woody plants, respectively. The state of each patch is characterized by its resource availability. We use a homogeneous environment here as all patches have the same, and constant, degree of resource limitation. One time step in the model represents approximately one year for woody plants and one day for non-woody plants.

**Table A1** State variables and initialization in the individual-based model. Actual values are drawn from the given intervals to introduce a certain degree of heterogeneity among individuals.

| **Variable** | **Description** | **Initial Value [unit] (woody/non-woody)** |
| --- | --- | --- |
| Plants |  |  |
| *c* | Initial growth rate | 1 ± 0.1 [kg m^-2^ time step^-1^] / [mg cm^-2^ time step^-1^] |
| *m_0_* | Initial body mass | 2 ± 0.2 [kg] / [mg] |
| *M_0_* | Maximal biomass | 2×10^6^ ± 2×10^5^ [kg] / [mg] |
| *m* | Current biomass | [kg] / [mg] |
| *A* | Zone of influence | [m^-2^] / [cm^-2^] |
| Patches |  |  |
| *RL* | Level of resource limitation | [0, 1] |
| Initialization |  |  |
| Mortality | Threshold of death | 2% of *m*^3/4^ |
| Density | Number of plants | 8100 ha^-1^ / m^-2^ |

*Process overview and scheduling*

After initialization, all individual plants with a given density are randomly distributed in the world. The processes of resource competition, growth and mortality of each plant are fulfilled within each time step. In each step, individual plants first sense the resource qualities of patches within their ZOIs, the area (radius) of an individual plant’s ZOI is determined by its current biomass. When their ZOIs are overlapping, individuals compete within the overlapping area. Thus, the overlapping area reflecting resources is divided according to the competition mode. Considering the outcome of the competition process, all individual plants grow according to the growth function. Plants with growth rates falling below a threshold die and are removed immediately. The state variables of the plants are synchronously updated within the subroutines, i.e. changes to state variables are updated only after all individuals have been processed (Grimm and Railsback 2005).

*Design concepts*

*Basic principles*: From “Metabolic Scaling Theory”, we derived a general ontogenetic growth model for individual plants. We combine this model, via the ZOI approach, with the effects of different modes of competition and resource limitation.

*Emergence*: All features observed at the population level, e.g. mass-density relationship or self-thinning trajectories (i.e. size distribution and spatial distribution, respectively), emerge from the interaction of individual plants with their neighbors and the resource level of their abiotic environment.

*Interaction*: Individual plants interact via competition for resources in the overlapping area of their ZOIs.

*Stochasticity*: Initial growth rate, initial biomass, maximum biomass and initial position of plants are randomly taken from the intervals given in Table A1. This introduces a certain level of heterogeneity among individual characteristics to take into account that real plants are never exactly identical.

*Observation*: Population size, biomass of each plant, and mean biomass of all living plants are the main observations.

*Initialization*

Initially, individual plants are randomly distributed according to the chosen initial density. Resources are spatially and temporally constant. Each plant has an initial biomass (*m*_0_), maximal biomass (*M*) and initial growth rate (*c*) drawn from truncated normal distributions with average and intervals given in Table 1.

*Input*

After initialization, the model does not include any external inputs, i.e. the abiotic environment is constant.

*Submodels*

*Plant growth*

In our individual-based model the plant’s ZOI stands for the physical space occupied by a plant and represents the energy and resources potentially available to this plant. This space is allometrically related to the plant’s body mass, *m*, as *c*_0_*A*=*m*^3/4^ (Enquist & Niklas 2001), where *c* is a normalization constant. Accordingly, we have:

*dm*/*dt* = *cA*[ 1 *–* ( *m* / *M*_0_ )^1/4^ ] (A1)

where *c* = *ac*_0_ is initial growth rates in units of mass per area and time interval. For simplicity, we choose 1 ± 0.1 in our model. We also simulate the model with different *c* values. As expected, the results from different values were qualitatively similar (the slopes did not change).

*Resource competition and limitation*

Resource limitation and competition usually cause a reduction of resource availability for plants. We therefore represent resource limitation via a dimensionless efficiency factor or index, *f_r_*, for different levels of resource availability. Resource competition is incorporated by using a dimensionless competition factor or index, *f_p_*, leading to

*dm*/*dt* = *f_r_**f_p_c**A*[ 1 *–* ( *m* / *M* )^1/4^ ] (A2)

where *M* = (*f_r_f_p_*)^4^*M*_0_, the maximum body size with resource limitation and competition.

The efficiency factor *f_r_*, can take different forms depending on the characteristics and level of the limiting resource. For simplification, we use a linear form here, i.e. *f_r_* =1*–RL*, where *RL* indicates the level of resource limitation, with its value ranging from 0 (no resource limitation) to 1 (maximum resource limitation; Table 1).

As for competition, the mode of resource-mediated competition among plants can be located somewhere along a continuum between completely asymmetric competition (largest plants obtain all the contested resources) and completely symmetric competition (resource uptake is equal for all plants, independent of their relative sizes; Schwinning & Weiner 1998). To represent different modes of competition explicitly, we describe the competitive factor *f_p_* as

$f_{p}=(A_{no}+\sum_{k=1}^{n_{o}} A_{o,k}\frac{m_{^{i}}^{p}}{\sum_{j=1}^{n_{j}} m_{j}^{p}})/A$ (A3)

this index refers to the fraction of resources available in the ZOI that the plant *i* can obtain after a loss of potential resources due to areas overlapped by *n_j_* individuals of sizes *m_j_* (Schwinning & Weiner 1998). *A_no_* is the area not overlapping with any neighbors, and *A_o,k_* indicates the area overlapped by neighbors. Parameter *p* determines the mode of competition, ranging from complete symmetry (*p* = 0) to complete asymmetry (*p* = ∞; for details and examples see Figure S1).


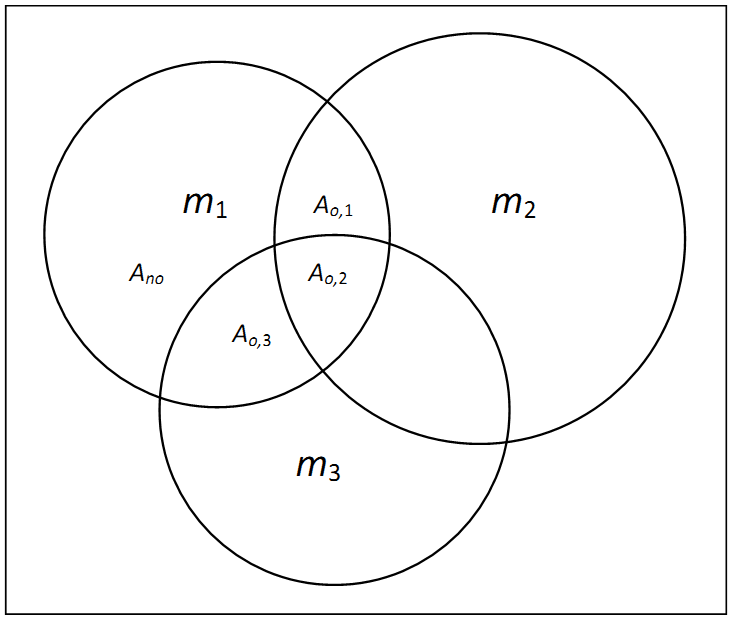


**Figure S1.** An example of calculating the competitive index (Eq. A3) with different modes of resource competition in an individual-based model as a way of dividing plants’ ZOI (zone-of-influence). Three plants with sizes *m*_1_, *m*_2_ and *m*_3_ are competing in this example. For plant 1, its ZOI (*A*) was divided into four parts: *A_no_*, the area not overlapping with the other two plants; *A_o_,*_1_, the area overlapping with plant 2; *A_o_,*_2_, the area overlapping with plants 2 and 3; *A_o_,*_3_, the area overlapping with plant 3.

Then the actual area that plant 1 can take from *A_o_,*_1_ is

$$A_{o,1}\frac{m_{1}^{p}}{\sum_{j=1}^{2} m_{j}^{p}}=A_{o,1}\frac{m_{1}^{p}}{m_{1}^{p}+m_{2}^{p}}$$

For *A_o_,*_2_,

$$A_{o,2}\frac{m_{1}^{p}}{\sum_{j=1}^{3} m_{j}^{p}}=A_{o,2}\frac{m_{1}^{p}}{m_{1}^{p}+m_{2}^{p}+m_{3}^{p}}$$

And for *A_o_,*_3_,

$$A_{o,3}\frac{m_{1}^{p}}{\sum_{j=1}^{2} m_{j}^{p}}=A_{o,3}\frac{m_{1}^{p}}{m_{1}^{p}+m_{3}^{p}}$$

Therefore, the competitive index for plant 1 is:

$$f_{p}=(A_{no}+A_{o,1}\frac{m_{1}^{p}}{m_{1}^{p}+m_{2}^{p}}+A_{o,2}\frac{m_{1}^{p}}{m_{1}^{p}+m_{2}^{p}+m_{3}^{p}}+A_{o,3}\frac{m_{1}^{p}}{m_{1}^{p}+m_{3}^{p}})/A$$

Where $A=m_{1}^{3/4}/c_{0}$

In total, equation (A2) clearly shows how a plant’s growth rate is jointly determined by resource availability, *f_r_*, and competition, *f_p_*. This also implies that a plant’s final size is usually smaller than its asymptotic maximum size (*M*) during resource limitation and local competition.

*Mortality*

An individual’s mortality rate is proportional to its mass-specific metabolism (Brow*n et* al. 2004). Based on this, we assume that individuals die if their actual growth rate (*dm*/*dt*) falls below a threshold of their current scaled body mass, i.e. 2% of *m*^3/4^. Therefore, individual plants may die due to metabolic inactivation driven by resource limitation, competition, senescence (when *m* approaches *M*) or combinations thereof. This provides a more realistic representation of relevant ecological process than in previous models (Stol*l et* al. 2002; Ch*u et* al. 2010).

**References**

Brown, J.H., Gillooly, J.F., Allen, A.P., Savage, V.M. & West, G.B. (2004). Toward a metabolic theory of ecology. *Ecology*, 85, 1771–1789.

Chu, C.J., Weiner, J., Maestre, F.T., Wang, Y.S., Morris, C., Xiao, S., et al. (2010). Effects of positive interactions, size symmetry of competition and abiotic stress on self-thinning in simulated plant populations. *Annals of Botany*, 106, 647–652.

Enquist, B.J. & Niklas, K.J. (2001). Invariant scaling relations across tree-dominated communities. *Nature*, 410, 655–660.

Grimm, V., Berger, U., Bastiansen, F., Eliassen, S., Ginot, V., Giske, J., et al. (2006). A standard protocol for describing individual-based and agent-based models. *Ecological Modelling*, 198, 115–126.

Grimm, V., Berger, U., DeAngelis, D.L., Polhill, J.G., Giske, J. & Railsback, S.F. (2010). The ODD protocol: A review and first update. *Ecological Modelling*, 211, 2760–2768.

Grimm, V. & Railsback, S.F. (2005). *Individual-based modeling and ecology*. Princeton University Press.

Schwinning, S. & Weiner, J. (1998). Mechanisms determining the degree of size asymmetry in competition among plants. *Oecologia*, 113, 447–455.

Stoll, P. & Bergius, E. (2005). Pattern and process: competition causes regular spacing of individuals within plant populations. *Journal of Ecology*, 93, 395–403.
